# EFFECT OF KINESIOLOGY TAPING ON LIMB OEDEMA IN PATIENTS WITH SEVERE HYPER-DEPENDENCY USING SHEAR-WAVE ELASTOGRAPHY: A RANDOMIZED CLINICAL TRIAL

**DOI:** 10.2340/jrm.v58.44783

**Published:** 2026-08-13

**Authors:** Qiumei LI, Liping LIN, Mengying FANG, Xiaodong TANG, Chuyao JIAN, Shaofeng ZHAO

**Affiliations:** Eighth Affiliated Hospital of Sun Yat-sen University, Shenzhen, China

**Keywords:** kinesiology taping, limb oedema, rehabilitation, severe hyper-dependency, shear-wave elastography

## Abstract

**Purpose:**

To determine the effects of kinesiology taping on reduced limb oedema using shear-wave elastography.

**Methods:**

A randomized trial with concealed allocation and intention-to-treat analysis. Fifty patients with limb oedema were randomly allocated to 2 groups: kinesiology taping at 25% tension applied to the oedematous tissue (25% kinesiology taping), and kinesiology taping at 0% tension applied to the oedematous tissue (0% kinesiology taping). The primary outcome was the elastic coefficient of shear-wave elastography (dermis and adipose layers), and the secondary outcome was limb circumference, which were assessed at days 0, 9, and 16.

**Results:**

Compared with the 0% kinesiology taping at day 9, the 25% kinesiology taping significantly reduced the elastic coefficient of the dermis layer (12.57 kPa, 95% CI 4.97 to 20.16, p = 0.001) and adipose layer (5.29 kPa, 95% CI 2.20 to 8.38, p < 0.001), and the length of limb circumference (2.18 cm, 95% CI 0.79 to 3.56, p < 0.001). The benefits of the dermis layer (p = 0.002) and adipose layer (p = 0.005), and the limb circumference (p < 0.001) were maintained at day 16. These findings demonstrate the sustained biomechanical improvements of oedematous tissues induced by 25% kinesiology taping, providing objective clinical evidence supporting the application of kinesiology taping for limb oedema management.

**Conclusion:**

Kinesiology taping at 25% tension reduces the elastic coefficient and limb circumference of limb oedema. We recommend kinesiology taping as a complementary, synergistic adjunct to limb oedema in patients with severe hyper-dependency.

## LAY ABSTRACT

Severely hyper-dependent patients often suffer from limbs edema, which interferes with their rehabilitation. Kinesiology tape is commonly used, but the benefit of applying the tape with stretch tension remained uncertain. We randomly assigned 50 patients with limb edema to receive either stretched (25% tension) or non‑stretched kinesiology taping. Limb circumference and edematous tissue stiffiness were followed for 16 days. Kinesiology tape applied with appropriate tension effectively reduced edema and softened edematous tissues, and these benefits persisted. This study supports using tension‑adjusted kinesiology taping as an extra rehabilitation measure for limb edema among patients with high care needs.

Limb oedema is a common complication in patients with severe hyper-dependency (1), commonly arising from factors like reduced physical activity, incorrect limb positioning, low serum albumin levels, renal failure, and cardiac insufficiency (2). The excess fluid accumulates in the interstitial space outside blood vessels, causing limb oedema, and visible depressions in the skin when minimal subcutaneous tissue is pressed with a finger (3). Limb oedema leads to pain, joint contractures, limited range of motion, amyotrophy and skin ulceration (4). These complications of limb oedema affect long-term recovery, causing increased hospitalization costs and extended hospital stays (5, 6).

Kinesiology tape (KT) (Performance Health, Akron, OH, USA) is a new technique of rehabilitation treatment (7), and it has been proposed for early-stage lower extremity oedema and pain (8, 9). This technique makes use of elastic adhesive tape, which is applied to the patient’s skin under tension, and can be extended up to 140% of its original length (7). This tape is a non-invasive and pharmaceutical-free elastic patch with a unique water-ripple acrylic adhesive surface similar to the elasticity of the skin, and does not restrict the range of motion (10, 11). The elastic tape can last for a period of 3–5 days and can be used in water (11). This technique applies unique materials to the skin surface, correcting joint dislocation, providing muscle support, activating the endogenous pain relief system, and promoting lymphatic reflux to eliminate oedema by changing the tensile force and cutting the shape according to the patient’s position to conform to the oedematous limb (7, 12).

KT is an intervention primarily designed to increase the space between the subcutaneous tissues, enhance local circulation, alleviate local inflammatory reactions, and reduce oedema. Although the effects of KT on limb oedema have been reported in patients after knee replacement arthroplasty (13-15) and in advanced cancer (16), the reported findings are inconsistent (17). One possible reason for the discrepancies is the wide range (10–75%) of KT tension reported across the studies. These data indicate that 10–15% tension of KT can reduce limb oedema and pain after knee-joint disease (8, 18). Several randomized trials have examined the functional effect of KT at 25–50% tension (19, 20). But increasing beyond 30% tension led to an immediate increase in pressure sensitivity (21). Recent studies have shown that KT applied at 25% tension is an optimal tension for reducing limb oedema and muscle pain in healthy adults (21, 22). However, the effects of KT at “optimal” tension on limb oedema in patients with severe hyper-dependency have not been determined.

Therefore, our research question for this randomized trial was: What are the relative effects of KT performed at 25% tension and 0% tension on limb oedema in patients with severe hyper-dependency? Specifically, we evaluated the efficacy of limb oedema using the SWE and Gulick anthropometric tape (Fabrication Enterprises Inc., Elmsford, NY, USA) anthropometric tape, comparing pre- and post-treatment in the elastic coefficient and LC between the 25% tension group and the 0% tension group over the post-treatment period.

## METHODS

### Study design

This prospective, two-arm, randomized trial was conducted with concealed allocation, blinded assessors, and intention-to-treat analysis. Patients who received rehabilitation for limb oedema at the Eighth Affiliated Hospital of Sun Yat-sen University, Shenzhen, China, between 8 December 2023 and 25 December 2024 and met the eligibility criteria were invited to our study.

Patients were randomly allocated to the KT at 25% tension group or the KT at 0% tension group according to a randomization scheme generated by computer. A physiotherapist who was not involved with the recruitment and treatment of patients randomly selected the blocks for each stratum and generated the final allocation sequence. The allocations were placed in sealed, opaque envelopes and stored in a concealed cabinet. When an eligible patient was recruited, the recruiting physiotherapist contacted the independent researcher to open the envelope and determine the patient’s group assignment. The intervention period was 9 days and the follow-up was 16 days, after which outcomes were measured and analysed. To maintain blinding, the physiotherapists delivering the KT intervention to different groups were conducted at separate times and in separate rooms. Outcome assessments were conducted by a different physiotherapist who was blinded to group allocation.

### Participants

The eligibility criteria are listed in Box 1. The physiotherapists were extensively trained to deliver the KT intervention and had a minimum of 3 years of clinical experience. All physiotherapists and researchers received standardized training before the trial to ensure procedure consistency.

Box 1 Eligibility Criteria

**Inclusion criteria:**

- Age >18 and <80 years
- Clinically diagnosed with severe hyper-dependency in line with the Guidelines for the capacity building and advancement of critical care medicine in China (23)
- Clinically diagnosed with limb oedema according to the Expert Consensus on the Diagnosis, Treatment, and Management of Oedema (24)
- The skin of the limb oedema is intact

**Exclusion criteria:**

- Involved in another research project or received another rehabilitation technique for treating limb oedema
- Serious cardiopulmonary conditions or any contraindications to the use of taping (such as skin allergy)
- An untreated ulcer or infection in the limb oedema
- Any condition that prevented the measurement or conduct of the study procedure

### Intervention

All patients from both groups received the KT reapplied in 3 sessions (3 consecutive days per session), making a total of 9 days. All patients might be discharged from the hospital, return home, or move to another rehabilitation hospital, but KT was discontinued after 9 days of intervention. Therefore, each participant received a total of 3 sessions over 9 days. Detailed procedures for the intervention are provided in a previous publication (25).

**Fig. 1:**
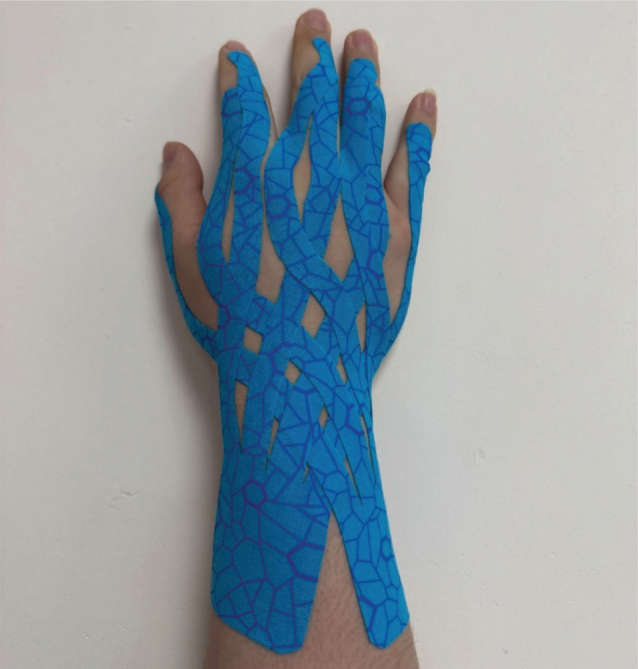
Image showing the specific application method of kinesiology taping.

*25% tension:* The KT used a fan-cut KT method, which was cut into a claw-shaped patch from one-third of the patient’s forearm to the middle finger. The application method employed a double lymphatic correction, based on the shape of the finger; each finger was attached using kinesiology tape and applied with a 25% elastic pulling force. Consequently, the glue surface was pushed from the proximal to the distal part of the finger and finally fixed to the tail end without force. Additionally, the 2 claw cloths were staggered to form an adhesive mesh as shown in Fig. 1. Two experienced physical therapists trained in kinesiology taping administered the interventions.

*0% tension:* Patients received the same KT method but without tension (i.e., 0% tension). The glue surface was pushed from the proximal to the distal part of the finger and finally fixed to the tail end without tension. Additionally, the 2 claw cloths were staggered to form an adhesive mesh.

### Outcome measures

The primary outcome was the elastic coefficient of the dermis and adipose layers, measured by the shear-wave elastography (SWE), as an indicator of limb oedema quantification. The secondary outcomes were limb circumference (LC), measured by the Gulick anthropometric tape. All outcomes were assessed at baseline (day 0), immediately after the 9-day intervention (day 9), and at the end of a 1-week follow-up period (day 16).

**Fig. 2:**
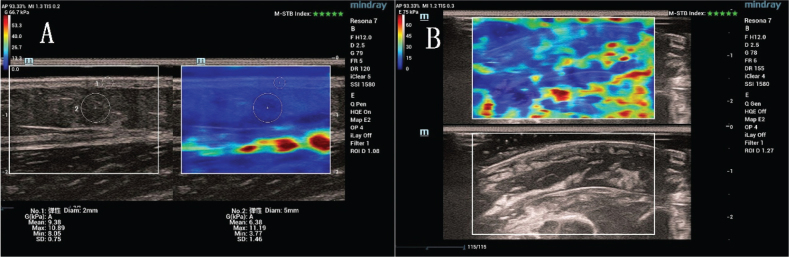
SWE images of skin and subcutaneous tissue. The SWE box was superimposed on the B-mode image to accurately depict the dermis and subcutis. The quantitative skin elastic moduli, including mean, minimum, maximum, and standard deviation, were automatically calculated by the Mindray system and displayed in the picture. (A) are SWE images of normal tissue, (B) SWE images of oedematous tissue.

*SWE:* SWE was performed by experienced sonographers using the Mindray Resona 7 (Mindray, China) with an SL15–4 multifrequency linear probe operating at 4–15 MHz. First, a square, coloured ROI was set at the targeted skin and subcutaneous tissue site for SWE data acquisition and superimposed on the B-mode image. The skin stiffness was displayed in colour within the ROI, as shown in Fig. 2A. Second, the superficial and deep margin of the ROI was manipulated to include the gel, skin, and subcutaneous tissue. During the SWE image acquisition, the probe was applied to the gel and maintained for several seconds to ensure that the practitioner obtained stable SWE images. Third, a small, round ROI was drawn within the first square ROI to quantitatively assess the skin and subcutaneous tissue stiffness. The parameters, expressed in kilopascal (kPa), were recorded for each oedematous tissue, as shown in Fig. 2B. The 5 consecutive SWE measurements were performed for each ROI to obtain the average values of the dermis layer and adipose layer. The operation was repeated 3 times, and the final average value of the dermis layer and adipose layer obtained.

*LC:* The LC was recorded using the Gulick anthropometric tape. The Gulick anthropometric tape was wrapped around the midpoint of the palm and the oedema circumference measured, without compressing the oedematous tissue. The LC parameters, expressed in centimetres (cm), were recorded for each oedematous tissue. The operation was repeated 3 times, and the final average value of the oedema circumference obtained.

### Statistical analyses

Continuous variables, including the elastic coefficient of SWE and LC, were expressed as means and standard deviations (SDs). Categorical variables, such as sex, were summarized using descriptive statistics. Multiple imputation (MI) was adopted to handle missing outcome data following the intention-to-treat (ITT) principle. Missing data accounted for 12% and were assumed to be missing at random (MAR). Ten imputed datasets were generated via an imputation model adjusted for demographics, disease duration, and baseline measurements. The analyses were performed on each imputed dataset separately, and pooled estimates were synthesized by Rubin’s rules as previously described (26–28). Baseline characteristic comparisons between groups were conducted to test randomization balance: one-way analysis of variance (ANOVA) and analysis of covariance (ANCOVA) were used for continuous variables, while the χ² test was adopted for categorical variables. Longitudinal outcome changes over day 0, day 9, and day 16 were evaluated via generalized estimating equations (GEE) with robust standard errors, with patient ID specified as the cluster variable and an exchangeable working correlation structure. The pre-specified primary confirmatory analyses were between-group comparisons of all indicators at day 9 and day 16. These 2 sets of core comparisons were regarded as the primary study hypotheses without additional multiple testing correction. Intra-group comparisons of day 9 vs day 0 and day 16 vs day 0 within each group were defined as post-hoc exploratory analyses, and no Bonferroni correction was applied to these exploratory pairwise comparisons.

All statistical tests were two-tailed, with the overall significance threshold set at α = 0.05. Data analysis was conducted using Statistical Package for the Social Sciences (SPSS) software for Windows, version 26 (IBM Corp, Armonk, NY, USA) and R software (version 4.4.1, R Foundation for Statistical Computing, Vienna, Austria). MedCalc software (version 10.4.7.0; MedCalc, Ostend, Belgium) was used to generate box-and-whisker plots. Longitudinal analytical results were reported as pooled mean differences, robust 95% confidence intervals (CIs), and corresponding raw *p*-values at each time interval.

### Sample size calculation

The required sample size was calculated by comparing the change from baseline to day 9 of intervention in the elastic coefficient using one-way analysis of variance. The standardized effect size was estimated to be 0.91 using our pilot data with preliminary experiment subjects, with a within-group SD of 0.81. With an alpha (α) of 0.05 and a power of 0.80 (β = 0.8), the estimated sample size was 22 participants per group. To account for 15% attrition, we increased the final required sample size to 25 participants per group (total *n* = 50).

### Sensitivity analysis

To evaluate the robustness of the findings, sensitivity analysis using distribution-free nonparametric complete case analysis (CCA) was conducted to examine whether the MI-GEE results stemmed from violated normality assumptions.

## RESULTS

**Fig. 3:**
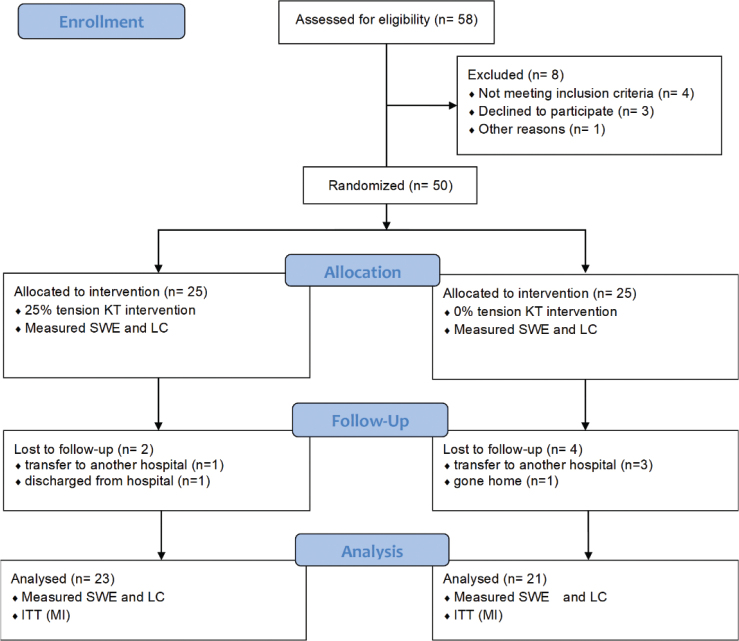
Design and flow of patients through the trial. SWE: shear wave elastography. LC: limb circumference. ITT: intention-to-treat. MI: multiple interpolation.

A total of 50 patients were randomized into 2 groups: 25% tension KT (*n* = 25) and 0% tension KT (*n* = 25). At day 9, 50 patients were assessed (100%). At day 16, 6 patients dropped out because of discharge and transfer to another hospital (25% tension: 2; 0% tension: 4). 44 patients were assessed (88% follow-up). The flow of patients through the trial is shown in Fig. 3. The baseline characteristics of the 50 included patients are presented in Table I. The random allocation process produced comparable groups at baseline.

**Table I:** Baseline and clinical characteristics

Clinical characteristics	25% tension KT (*n* = 25)	0% tension KT (*n* = 25)	*p*-value
Age (years), mean (SD)	66.5 (10.5)	65.8 (9.9)	0.719
Sex, *n* (%)			0.763
Female	10 (40)	11 (44)	
Male	15 (60)	14 (56)	
Comorbidities, *n* (%)			
Low protein	23(92)	22(84)	0.637
Cardiac insufficiency	11(44)	14(56)	0.396
Renal insufficiency	7(28)	9(36)	0.544
Use diuretic	19(76)	22(88)	0.269
Time bedridden (days), *n* (%)			0.848
< 2 weeks	5 (20)	4 (16)	
2–4 weeks	13 (52)	15 (60)	
> 4 weeks	7 (28)	6 (24)	
Therapy site, *n* (%)			0.490
EICU	9 (36)	13 (52)	
RICU	5 (20)	3 (12)	
ICU	11 (44)	9 (36)	
Principal diagnosis, *n* (%)			0.601
Cerebrovascular disease	15 (60)	17 (68)	
Heart disease	2 (8)	3 (12)	
Lung disease	8 (32)	5 (20)	
Elastic coefficient of SWE (kPa), mean (SD)			
Dermis layer	31.4 (17.4)	28.9 (19.5)	0.479
Adipose layer	19.5 (7.5)	17.7 (7.9)	0.399
LC (mm), mean (SD)	23.7 (2.4)	23.6 (2.3)	0.954

EICU: emergency intensive care unit; ICU: intensive care unit; RICU: respiratory intensive care unit; SWE: shear wave elastography; TDU: two-dimensional ultrasound; LC: limb circumference.

a Values are represented as numbers (%) unless otherwise stated. Means and standard deviations (SDs) are based on the total baseline sample.

### Primary outcomes

**Fig. 4:**
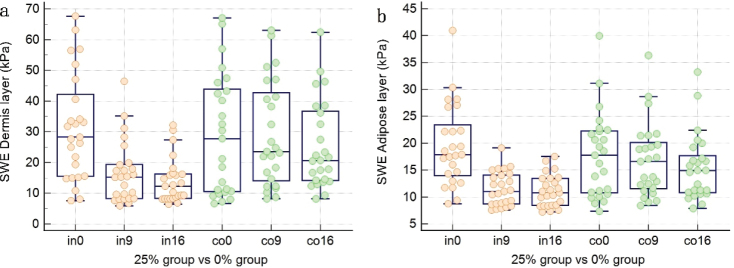
(A) Box plot of SWE-dermis layer elasticity coefficient between the 25% tension group and the 0% tension group. (B) Box plot of SWE-adipose layer elasticity coefficient between the 25% tension group and the 0% tension group.

Compared with the 0% tension KT group, 9 days of 25% tension KT reduced the elastic coefficient of the dermis layer by 12.57 kPa (95% CI 4.97 to 20.16, *p* = 0.001). The elastic coefficient of the adipose layer reduced the same outcomes by 5.29 kPa (95% CI 2.20 to 8.38, *p* < 0.001). At day 16, all the benefits of 25% tension KT were maintained. 25% tension KT was clearly more beneficial than 0% tension KT on all elastic coefficients of the dermis layer (95% CI 4.12 to 18.73, *P* = 0.002) and adipose layer (95% CI 1.33 to 7.25, *p* = 0.005). The effect sizes indicating the actual between-group difference are highly substantial with strong clinical and practical significance (Table II). The comparison of the results is shown in Fig. 4.

**Table II:** Adjusted mean (95% CIs) difference and effect size between groups

Item	Pairwise comparison, AMD (95 % CI*s*), Bonferroni-adjusted *p-*value					
	25% tension KT (*n* = 25) vs 0% tension KT (*n* = 25)					
	SWE (kPa)				LC (cm)	
	Dermis layer	*p-*value	Adipose layer	*p-*value		*p-*value
9 days	12.57(4.97, 20.16)	0.001	5.29(2.20, 8.38)	< 0.001	2.18(0.79, 3.56)	< 0.001
16 days	11.43(4.12, 18.73)	0.002	4.29(1.33, 7.25)	0.005	2.63(1.44, 3.83)	< 0.001
ES	1.92^a^ 0.87^b^		1.01^a^ 0.80^b^		1.01^a^ 1.22^b^	

AMD: adjusted mean difference; CI: confidence interval; LC: limb circumference; *p*-value: Bonferroni-adjusted *p-*value; SWE: shear-wave elastography; KT: kinesiology taping; ES: effect size, use of Cohen’s d;

a effect size at day 9 between groups;

b effect size at day 16 between groups.

### Secondary outcome

**Fig. 5:**
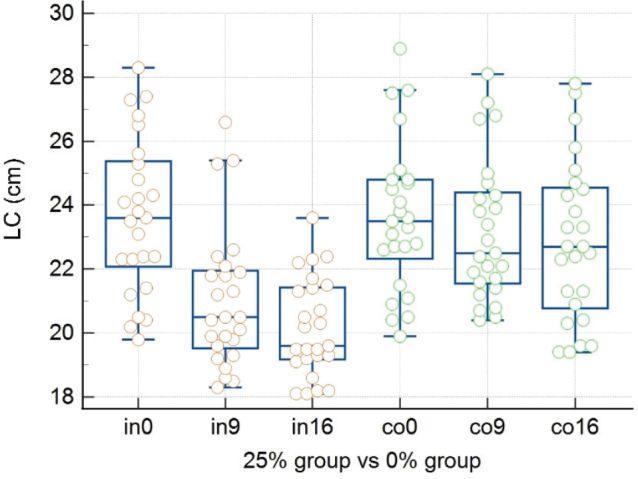
Box plot of LC between the KT group and the control group.

Compared with the 0% tension KT group, 9 days of 25% tension KT reduced LC by a mean of 2.18 cm (95% CI 0.79 to 3.56, *p* < 0.001), and this effect was largely maintained at 16 days (95% CI 1.44 to 3.83, *p* < 0.001). The effect sizes indicate that the actual between-group difference is substantial, with strong clinical and practical significance (see Table II). The comparison of the results is shown in Fig. 5.

Intra-group comparisons between each follow-up time point and baseline were exploratory post-hoc analyses without multiple testing correction. All indicators presented a decreasing trend over time in the experimental group, whereas no notable temporal variations were detected in the control group. Detailed statistical results are available in Table III.

**Table III:** Adjusted mean (robust 95% CIs) difference within groups

Item	Change from baseline, AMD (95 % CI)					
	25% tension KT (*n* = 25)			0% tension KT (*n* = 25)		
	SWE		LC	SWE		LC
	Dermis layer	Adipose layer		Dermis layer	Adipose layer	
9 days	14.76(9.56, 19.97)	8.00(5.21, 10.80)	2.62(1.68, 3.57)	0.27(–3.13, 3.68)	–0.89(–2.53, 0.74)	–0.43(–1.06, 0.19)
16 days	16.49(10.81, 22.17)	8.05(5.23, 10.86)	3.48(2.73, 4.23)	–2.60(–2.62, 7.81)	–1.94(–4.58, 0.70)	–0.83(–2.14, 0.48)
*P*_bonferroni_	<0.001^a^ <0.001^b^	<0.001^a^ <0.001^b^	<0.001^a^ <0.001^b^	0.875^a^ 0.325^b^	0.282^a^ 0.149^b^	0.176^a^ 0.212^b^

AMD: adjusted mean difference; CI: confidence interval; LC: limb circumference; SWE: shear-wave elastography; KT: kinesiology taping;

a *p*-value between day 9 and the baseline;

b *p*-value between day 16 and the baseline.

### Sensitivity analysis

Sensitivity analyses confirmed that the direction and magnitude of KT effects were consistent with the intention-to-treat analysis, and statistical significance was largely maintained, indicating that the main findings were robust despite the missing data (Table IV).

**Table IV:** Difference between groups after sensitivity analysis

Analysis	Mean (SD) 25% tension vs 0% tension		*p-*value
	9-day	16-day	
MI-GEE (MAR-PMM)			
Dermis layer	16.62(10.02) vs 29.19(17.05)	14.89(8.30) vs 26.32 (14.83)	0.001^a^ <0.001^b^
Sdipose layer	11.50(3.15) vs 16.79(6.87)	11.46(3.33) vs 15.75(6.29)	<0.001^a^ 0.002^b^
LC	21.04(2.18) vs 23.21(2.22)	20.18(1.58) vs 22.81(2.48)	0.005^a^ < 0.001^b^
Complete case analysis (CCA)			
Dermis layer	16.61 (10.46) vs 31.02 (17.38)	14.27 (7.52) vs 26.65 (15.12)	0.002^a^ 0.002^b^
Adipose layer	11.44 (3.16) vs 17.53 (7.19)	11.27 (3.06) vs 16.02 (6.43)	0.001^a^ 0.005^b^
LC	20.96 (2.25) vs 23.08 (2.21)	20.15 (1.58) vs 22.90 (2.51)	0.001^a^ <0.001^b^

MI-GEE: Multiple Imputation combined with Generalized Estimating Equations; CCA: complete case analysis; SD: standard deviation; CI: confidence interval; LC: limb circumference; MI: multiple imputation; MAR: missing at random; PMM: pattern mixed model; KT: kinesiology taping;

a *p-*value between day 9 and the baseline;

b *p-*value between day 16 and the baseline.

### Safety

In our study, no serious adverse events related to the intervention were observed. Two cases of reddening of skin in the 25% tension KT group were reported, and 1 occurred in the 0% tension KT group. There was no significant difference between the groups (*p* = 0.862; Table V).

**Table V:** Adverse events related to treatment^a^

Item	Kinesiology taping group (*n* = 25)	Sham Kinesiology taping group (*n* = 25)
Overall*	2(8.7)	1(4.8)
Serious adverse event	0(0)	0(0)
Adverse event	2(8.7)	1(4.8)
Rubefaction	2(8.7)	1(4.8)
Skin allergy	0(0)	0(0)
Skin inflamed	0(0)	0(0)
Skin lacerations	0(0)	0(0)

Data are *n* (%).

a An adverse event with multiple occurrences in a single participant was defined as 1 adverse event.

* Fisher’s exact test was used to analyse adverse events between the 2 groups (*p* = 0.862).

## DISCUSSION

Our study aimed to compare the training efficacy of a KT protocol at 25% tension in people with severe hyper-dependency, using 0% tension KT as a control group. The results showed that 25% tension KT significantly reduced the elastic coefficient of dermis and adipose layers and LC compared with the 0% tension KT group. Subsequently, these benefits were maintained during the 16-day post-intervention follow-up, with the greatest improvements consistently observed in the 25% tension KT group.

In agreement with other reports, 25% tension KT treating reduced limb oedema (19). Horoz et al. reported similar benefits with KT over 10 days in patients with distal radius fractures, showing reductions in arm and trunk oedema (25). Several randomized trials illustrated that 10–15% tension of KT can reduce limb oedema after knee-joint disease (8, 18). Notably, Huang et al. suggested that 15% tension KT did not reduce the limb oedema in patient with stroke (12). The improvement in SWE and LC observed in our study may be explained by the training intensity of 25% tension KT because it has been reported that limb oedema at 25% tension KT is associated with the greatest increase in the space between the subcutaneous tissues, with enhancement of local circulation and alleviation of local inflammatory reactions (21). The greater improvement with 25% tension KT may also be due to increased superficial fascia activation and provided circulatory/lymphatic correction, and mechanical support (29–31).

Peripheral compression in addition to muscle facilitation techniques of KT had positive impacts on limb oedema (32). The positive effects of compression stockings on oedema has been reported in patients with limb oedema (33, 34). Previous studies emphasizing benefits of peripheral compression showed that mixed a KT compression model reduced limb oedema (35, 32). In addition, some data that compared the effects of KT and compression therapy in postmastectomy lymphoedema patients found no significant difference between KT and compression therapy for limb oedema (36, 37). In particular, the study by Tantawy et al. demonstrated that KT resulted in significant amelioration of limb oedema in comparison with compression garments 3 weeks post treatment (38). Several studies have suggested that the fan-shaped technique of KT was better than compression therapy for pain and oedema reduction in patients with breast-cancer-related lymphoedema (38, 39). Tantawy and Pajero Otero et al. claimed that compression therapy failed to decrease oedema as efficiently as fan-shaped technique of KT due to low duration of usage of compression garments and pressure changes in compression garments in time (38, 39). Therefore, the findings of our study are in accordance with these results.

The primary outcome, SWE, is a two-dimensional performance-based assessment tool that provides insight into the dynamic balance dimensions of limb oedema (40). The SWE can also be used to monitor the progression of oedema tissue during therapy (41). Comparative studies of SWE and oedema extent showed that oedematous tissue directly correlates with the thickness and stiffness (42). The SWE can assess the increased thickness and stiffness of oedematous tissue and pressure within the tissues due to tissue oedema. Consequently, SWE parameters appear valuable for evaluating limb oedema in patients with severe hyper-dependency. Future studies can utilize SWE to assess the dynamic changes in oedema pre- and post-treatment.

The deterioration in physical quality of life in patients with severe hyper-dependency is more associated with a complicated condition than the existence of limb oedema. However, despite this, KT treatment improved the physical dimension of the quality of life through reducing pain and limb oedema, due to greatest increase in the space between the subcutaneous tissues, enhanced local circulation, and alleviated local inflammatory reactions. Therefore, for hyper-dependency patients, KT techniques increased superficial fascia activation and provided circulatory/lymphatic correction, and mechanical support of oedematous tissue. This kind of support of limb oedema probably enabled improvement in functional capacity and thus quality of life for the patients.

### Limitations

Some (12%) of our cohort failed to present at the 16-day follow-up assessment. This confounded our evaluation of the sustained effect of KT at day 16. However, despite this, we were able to demonstrate that the SWE and LC of our remaining cohort were maintained at 16 days beyond the end of the intervention. These findings support KT in rehabilitation protocols for patients with limb oedema with severe hyper-dependency. Further studies on long-term and continuing KT effects are warranted.

### Conclusion

In conclusion, KT performed at 25% tension significantly reduced the elastic coefficient of dermis and adipose layers and the LC. Treating on a 25% tension confers larger magnitude benefits over KT on a 0% tension and reduced the limb oedema. Many of these benefits are maintained for up to 16 days beyond the intervention period. We recommend early application of KT as part of a multidisciplinary approach to managing limb oedema in patients with severe hyper-dependency. Future larger-scale trials are warranted to confirm its effectiveness and investigate the underlying mechanisms.
